# Involvement of midkine in the development of pulmonary fibrosis

**DOI:** 10.14814/phy2.13383

**Published:** 2017-08-15

**Authors:** Kenichi Misa, Yoshinori Tanino, Xintao Wang, Takefumi Nikaido, Masami Kikuchi, Yuki Sato, Ryuichi Togawa, Mishie Tanino, Shinya Tanaka, Kenji Kadomatsu, Mitsuru Munakata

**Affiliations:** ^1^ Department of Pulmonary Medicine Fukushima Medical University School of Medicine Fukushima Japan; ^2^ Department of Cancer Pathology Hokkaido University Graduate School of Medicine Sapporo Japan; ^3^ Department of Biochemistry Nagoya University School of Medicine Nagoya Japan

**Keywords:** Inflammation, midkine, pulmonary fibrosis, TGF‐*β*

## Abstract

Midkine is a low‐molecular‐weight heparin‐binding protein that is strongly expressed mainly in the midgestation period and has various physiological activities such as in development and cell migration. Midkine has been reported to be strongly expressed in cancer cells and in inflammation and repair processes, and to be involved in the pathogenesis of various diseases. However, its role in the lung is poorly understood. In this study, we analyzed the clinical characteristics of idiopathic pulmonary fibrosis patients in relation to midkine expression and used a mouse bleomycin‐induced pulmonary fibrosis model to investigate the role of midkine in pulmonary fibrosis. In the idiopathic pulmonary fibrosis patients, the serum midkine level was significantly higher than in healthy subjects, and midkine levels in the serum and bronchoalveolar lavage (BAL) fluid correlated positively with the percentage of inflammatory cells in the BAL fluid. In wild‐type mice, intratracheal bleomycin administration increased midkine expression in lung tissue. Additionally, compared with wild‐type mice, midkine‐deficient mice showed low expression of both collagen and *α*‐smooth muscle actin, as well as a low value for the pathological lung fibrosis score after bleomycin administration. Furthermore, the total cell count and lymphocyte percentage in the BAL fluid, as well as TNF‐*α* and transforming growth factor‐*β* expression in lung tissue, were significantly lower in the midkine‐deficient mice compared with wild‐type mice. These results suggest that midkine is involved in the development of pulmonary fibrosis by regulating inflammatory cell migration into the lung, and TNF‐*α* and transforming growth factor‐*β* expression.

## Introduction

Idiopathic pulmonary fibrosis (IPF) is an intractable lung disease of unknown cause that progresses to severe lung fibrosis, with a mean survival time of only 3–5 years from diagnosis (American Thoracic Society, 2000). The annual incidence rate and prevalence of IPF in Japan are 2.5 and 11.0 per 100,000 population, respectively, and it is estimated that more than 13,000 patients are affected (Natsuizaka et al. 2014). The mechanism of pulmonary fibrosis is complex, and various types of cells are involved. Pulmonary fibroblasts play an important role in pulmonary fibrosis by producing excessive ECM and differentiating into myofibroblasts. However, there are still many unclear points regarding the full picture of the molecular pathophysiology of pulmonary fibrosis.

Midkine, discovered in 1988, is a low‐molecular‐weight heparin‐binding protein with a molecular weight of about 13 kDa (Kadomatsu et al. 1988). It is strongly expressed mainly in epithelial tissues in the process of epithelial mesenchymal interactions, in differentiating nerve tissues, and in mesoderm undergoing remodeling. Midkine also has various physiological activities such as roles in development, survival and cell migration (Kadomatsu et al. 1988, 1990; Matsubara et al. 1990). Expression of midkine in adults is limited, but it has been reported to be strongly expressed in cancer cells (Tsutsui et al. 1993) and the processes of inflammation and repair (Ohta et al. 1999), and to be involved in the pathology of various diseases (Muramatsu 2002).

There are few reports dealing with the role of midkine in the lung. However, it has been reported that its expression is accelerated in the respiratory tract of cystic fibrosis patients (Nordin et al. 2013) and that it has antimicrobial activity against bacteria and fungi (Noble et al. 2012; Nordin et al. 2012; Linge et al. 2013; Netsu et al. 2014; Nikaido et al. 2015). While these results suggest that midkine plays an important role in pulmonary inflammation, Zhang et al. (2015) has recently reported the role of midkine in acute respiratory distress syndrome (ARDS)‐associated lung fibrosis. However, the role of midkine in lung diseases remains unclear in detail.

To determine the role of midkine in pulmonary fibrosis, we analyzed the clinical characteristics of patients with IPF and used a mouse bleomycin‐induced pulmonary fibrosis model in this study.

## Materials and Methods

### Analysis in IPF patients

Patients with IPF admitted to our department with no progression of symptoms such as dyspnea and radiological findings for at least 3 months were analyzed retrospectively. Serological tests, pulmonary function tests, high‐resolution computed tomography and bronchoalveolar lavage (BAL) were performed for these patients. The diagnosis of IPF was made in accordance with the 2011 ATS/ERS/JRS/ALAT joint statement (Sato et al. 2017). Midkine concentrations in serum and BAL fluid were measured using the Human Midkine DuoSet ELISA kit (R&D; Minneapolis, MN) and were examined for associations with the clinical characteristics of the IPF patients (Tables 1 and 4a). Serum samples were also obtained from healthy subjects. Both IPF patients and healthy subjects granted informed consent. This study was conducted with the approval of the Ethics Committee of Fukushima Medical University.

**Table 1:** Clinical characteristics of healthy volunteers and patients with IPF

	Healthy volunteers	IPF
Subjects (*n*)	10	40
Age (years)	32.6 ± 2.4	69.5 ± 1.1
Gender (M/F)	4/6	34/6
WBC (/*μ*L)	N/A	7590 ± 491
LDH (U/mL)	N/A	237 ± 10
CRP (mg/dL)	N/A	0.6 ± 0.2
ESR (mm/h)	N/A	20 ± 3
KL‐6 (U/mL)	N/A	1251 ± 111
SP‐A (ng/mL)	N/A	107.2 ± 13.5
SP‐D (ng/mL)	N/A	241.2 ± 26.8
PaO_2_ (mmHg)	N/A	83.3 ± 1.9
*P*/*F* (mmHg)	N/A	394.3 ± 10.5
%VC (%)	N/A	82.8 ± 2.8
%DL_CO_ (%)	N/A	57.0 ± 3.3

Mean ± SEM. IPF, idiopathic pulmonary fibrosis, M, Male, F, female, WBC, white blood cell, LDH, lactate dehydrogenase, CRP, C‐reactive protein, ESR, erythrocyte sedimentation ratio, KL‐6, Krebs von den Lungen‐6, SP‐A, surfactant protein‐A, SP‐D, surfactant protein‐D, PaO_2_, partial pressure of arterial oxygen, *P*/*F*, partial pressure of arterial oxygen/inspired oxygen fraction, VC, vital capacity, DL_CO_, diffusing capacity of the lung carbon monoxide, N/A, not available.

### Mouse bleomycin pulmonary fibrosis model

Midkine‐deficient (*Mdk* KO mice; Dr. K. Kadomatsu) mice used in these experiments have no gross abnormalities by macroscopic and microscopic observation in the brain, lung, heart, stomach, kidney, testis or ovary as Nakamura et al. (1998) previously reported. Wild‐type (WT) and *Mdk* KO mice were anesthetized with ketamine/xylasine, and 2.0 mg/kg of bleomycin hydrochloride (Nippon Kayaku; Tokyo, Japan) was administered intratracheally. BAL was performed as previously described (Strieter and Mehrad 2009; Netsu et al. 2014). Briefly, a BD insyte autoguard (Becton, Dickinson and Company; NJ) catheter was inserted into the trachea and 0.6 mL of physiological saline was infused a total of three times. After recovery of the fluid, the mice were sacrificed and excised lung tissue was used to analyze the mRNA expression of mediators. In addition, for histopathological examinations, 10% formalin (Wako Pure Chemical Industries; Osaka, Japan) was injected into the trachea and fixed with 25 cm H_2_O at 14 days after bleomycin administration.

### Measurement of mediator mRNA in mouse lung tissue

RNA was then extracted from the harvested mouse lung tissue with an Absolutely RNA Miniprep Kit (Stratagene; La Jolla, CA) as reported previously (Strieter and Mehrad 2009; Netsu et al. 2014). DNase I was used to digest genomic DNA, and RNA was reverse‐transcribed using a SuperScript III First‐Strand Synthesis System (Invitrogen; Carlsbad, CA). Quantitative real time PCR was performed using the Power SYBR Green PCR Master Mix and the ABI PRISM 7000 (Applied Biosystems; Foster City, CA) with the primers shown in Table 2. The threshold cycle was calculated using threshold cycles for the target genes and 18S. Relative mRNA expression was expressed as fold increase over values obtained from RNA from mouse normal lungs.

**Table 2:** Primers for quantitative real‐time PCR

Midkine	(F):5′‐CTCGCCCTTCTTGCCCTCTT‐3′ (R):5′‐GCAGGGCACCTTGCAATGGA‐3′
TNF‐*α*	(F):5′‐GACCCTCACACTCAGATCATCTTC‐3′ (R):5′‐CGCTGGCTCAGCCACTCC‐3′
KC	(F):5′‐GCTCGCTTCTCTGTGCAG‐3′ (R):5′‐GGAGCTTCAGGGTCAAGG‐3′
MIP‐2	(F):5′‐AAGTCATAGCCACTCTCAGG‐3′ (R):5′‐AGCGAGGCACATCAGGTAC‐3′
Collagen I	(F):5′‐TGTTGGCCCATCTGGTAAAGA‐3′ (R):5′‐CAGGGAATCCGATGTTGCC‐3′
*α*‐SMA	(F):5′‐CTGCCGAGCGTGAGATTG‐3′ (R):5′‐ATAGGTGGTTTCGTGGATGC‐3′
TGF‐*β*	(F):5′‐ CCATCCATGACATGAACCGA ‐3′ (R):5′‐ CAGGTGTTGAGCCCTTTCCA ‐3′
GAPDH	(F):5′‐CATGGTCTACATGTTCCAGT‐3′ (R):5′‐GGCTAAGCAGTTGGTGGTGC‐3′

(F), forward; (R), reverse.

### Assay of protein concentrations in BAL fluids

Protein concentrations in mouse BAL fluids were determined using a BCA Protein Assay Kit (Thermo Fisher Scientific; Rockford, IL).

### Assay of collagen content in lung tissue

Collagen content in mouse lung tissue was measured using the Sircol Collagen Assay Kit (Biocolor; Antrim, UK). Briefly, lung tissue homogenates in 0.5 mol/L acetic acid (Sigma‐Aldrich; St. Louis, MO) containing 0.1% pepsin (Sigma‐Aldrich) were stirred slowly overnight on a shaker, and assays were performed according to the manufacturer's protocol.

### Histopathological assessment of pulmonary fibrosis

Formalin‐fixed mouse lungs were embedded in paraffin, sliced in a thickness of 4 *μ*m, and stained with hematoxylin and eosin. Pulmonary fibrosis was assessed using the Ashcroft score, as previously reported (Tanino et al. 2002). In this assessment, the score was ranked into nine grades, from Grade 0 to 8. All animal experiments were performed with the approval of the Animal Experiments Committee of Fukushima Medical University.

### Statistics

The Mann–Whitney *U* test was used to compare two groups, while ANOVA with Fisher's least significant difference test was used to compare multiple groups. Pearson's correlation coefficient was used for correlation analysis. The analysis was performed using IBM SPSS Statistics 17.0 software (IBM Japan; Tokyo, Japan), and *P* < 0.05 was considered statistically significant.

## Results

### Serum midkine concentration and clinical characteristics of IPF patients

**Figure 1:**
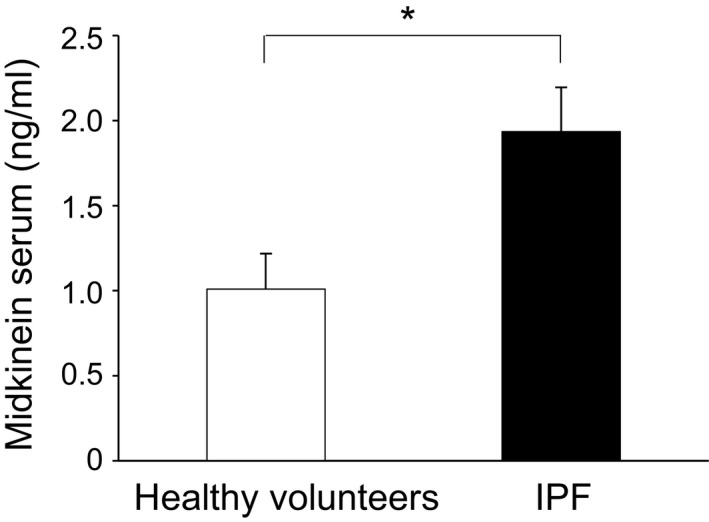
Serum midkine concentrations in healthy subjects and IPF patients. Serum midkine concentrations in healthy volunteers (*n* = 10) and IPF patients (*n* = 40) were compared using the Mann–Whitney *U* test. *P < 0.05 versus healthy volunteers.

Serum midkine levels were compared between 40 IPF patients and 10 healthy subjects and were found to be significantly higher in the IPF patients (Fig. 1). Also, correlations between the serum midkine concentration and clinical characteristics of IPF patients were investigated (Table 3). Although the serum midkine concentration in the IPF patients showed no associations with any of the blood test or pulmonary function test parameters, it correlated positively with the percentage of neutrophils in the BAL fluid, and tended to show positive correlations with the percentage of eosinophils and lymphocytes. Moreover, the midkine concentration in the BAL fluid (472.9 ± 43.3 pg/mL) in IPF patients showed a trend to a positive correlation with the serum midkine concentration (*r* = 0.365, *P* = 0.067). The midkine concentration in the BAL fluid showed a positive correlation with the percentage of eosinophils in the BAL fluid, and tended to correlate positively with the percentage of lymphocytes and neutrophils, although there was no correlation between these concentrations and parameters of the blood tests or pulmonary function test (Table 4b).

**Table 3:** Correlation between serum midkine and clinical parameters in patients with IPF

	Correlation coefficients	*P* value
WBC (/*μ*L)	−0.010	0.960
LDH (mg/dL)	0.313	0.105
CRP (mg/dL)	0.279	0.151
ESR (mm/h)	0.203	0.300
KL‐6 (U/mL)	0.003	0.988
SP‐A (ng/mL)	−0.013	0.948
SP‐D (ng/mL)	0.040	0.838
*P*/*F* (torr)	0.088	0.655
Lym in BAL fliud (%)	0.351	0.079
Neu in BAL fliud (%)	0.481	0.013
Eos in BAL fliud (%)	0.368	0.065
%VC (%)	0.076	0.701

*N* = 40. WBC, white blood cell, LDH, lactate dehydrogenase, CRP, C‐reactive protein, ESR, erythrocyte sedimentation ration, KL‐6, Krebs von den Lungen‐6, SP‐A, surfactant protein‐A, SP‐D, surfactant protein‐D, *P*/*F*, partial pressure of arterial oxygen/inspired oxygen fraction, Lym, lymphocyte, Neu, neutrophil, Eos, eosinophil, BAL, bronchoalveolar lavage, VC, vital capacity.

**Table 4:** Correlation between midkine in BAL fluid and clinical parameters in patients with IPF

(a) BAL fluid findings					
TCC (×10^4^/mL)	AM (%)	Lym (%)	Neu (%)	Eos (%)	CD4/CD8
24.8 ± 2.7	77.0 ± 3.3	9.9 ± 1.3	8.4 ± 1.9	3.2 ± 0.8	1.9 ± 0.3

(b) Correlation between midkine in BAL fluid and clinical parameters		
	Correlation coefficients	*P* value
Lym in BAL fliud (%)	0.292	0.080
Neu in BAL fliud (%)	0.274	0.101
Eos in BAL fliud (%)	0.383	0.019
%VC (%)	0.017	0.922

*N* = 40. Mean ± SEM. TCC, total cell count, AM, alveolar macrophage, Lym, lymphocyte, Neu, neutrophil, Eos, eosinophil; BAL, bronchoalveolar lavage, %VC, vital capacity.

### Change in midkine expression in mouse lung tissue after bleomycin administration

**Figure 2:**
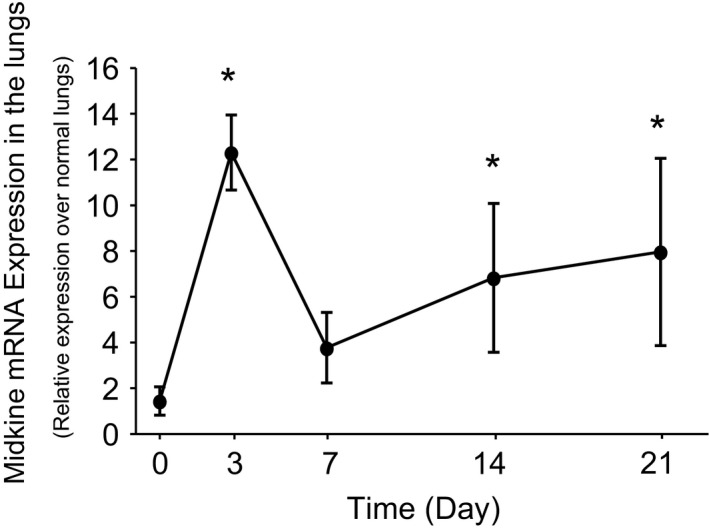
Expression of midkine in mouse lung tissue after intratracheal bleomycin administration. Midkine mRNA concentration in the lung tissue of mice on the indicated days after bleomycin administration is shown (*n* = 4–5 per time point). Statistical differences between each group and Day 0 were compared using ANOVA with Fisher's least significant difference test as a post hoc test. *P < 0.05 versus Day 0.

To investigate the role of midkine in bleomycin‐induced pulmonary fibrosis, we first analyzed the time‐course of midkine mRNA expression in mouse lung tissue after intratracheal bleomycin administration. Midkine mRNA expression in mouse lung tissue was significantly increased at 3 days after bleomycin administration, but it had decreased at 7 days and then gradually increased again from 14 days onward (Fig. 2).

### Role of midkine in bleomycin‐induced pulmonary fibrosis

Next, WT and *Mdk* KO mice were used to investigate the role of midkine in pulmonary fibrosis as follows.

### Analysis of pulmonary fibrosis

**Figure 3:**
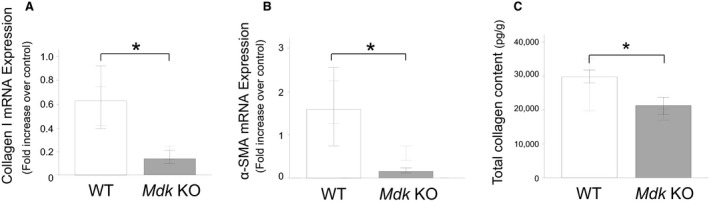
Expression of type I collagen and *α*‐ SMA in lung tissue and total collagen content in lung tissue at 14 days after intratracheal bleomycin administration. Expression of type I collagen (A) and *α*‐SMA (B) mRNA as well as total collagen content (C) in the lung tissue of midkine‐deficient (*Mdk* KO;* n* = 8–9) and wild‐type (WT;* n* = 8–14) mice at 14 days after bleomycin intratracheal administration was compared using the Mann–Whitney *U* test. *P < 0.05 versus *Mdk* KO.

**Figure 4:**
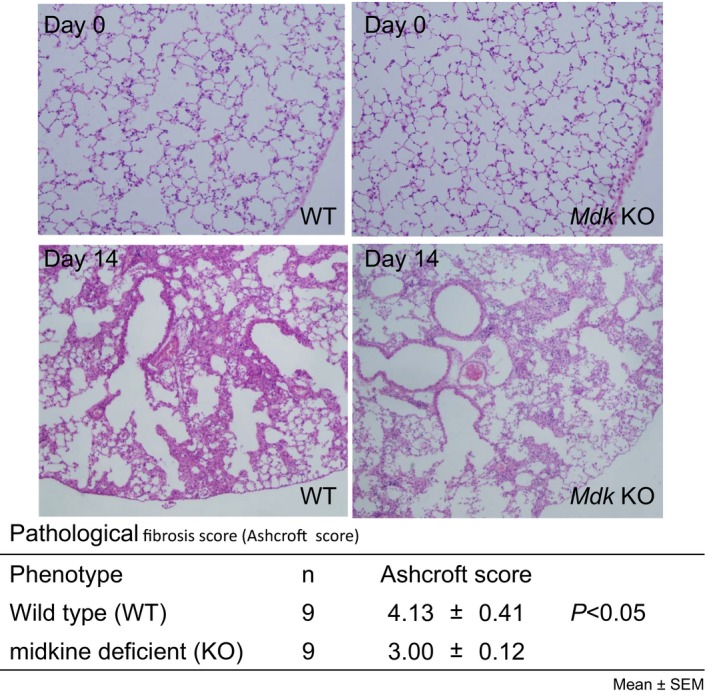
Histopathological findings before and at 14 days after intratracheal bleomycin administration. Pathological lung fibrosis score of midkine‐deficient and wild‐type mice (*n* = 9 per each group) at 14 days after bleomycin intratracheal administration was compared using the Mann–Whitney *U* test. Pictures of H&E staining (upper panel) are representatives 4 and 9 mice for before and at 14 days, respectively.

At 14 days after bleomycin administration, *Mdk* KO mice showed, in comparison with WT mice, significantly lower expression of mRNA for both type I collagen and *α*‐smooth muscle actin (*α*‐SMA) as well as a significantly lower collagen content in the lung tissue (Fig. 3). Also, the pathological fibrosis score was significantly lower in the *Mdk* KO than in the WT mice, although there was no different pathological findings between the *Mdk* KO and WT mice before bleomycin administration (Fig. 4). These results suggest that bleomycin‐induced lung fibrosis was suppressed in the *Mdk* KO mice.

### Analysis of BAL fluid

**Figure 5:**
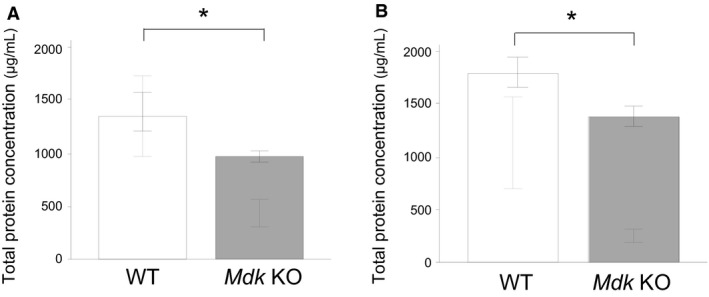
Total protein concentration in bronchoalveolar lavage fluid findings at 7 and 14 days after intratracheal bleomycin administration. Total protein concentration in bronchoalveolar lavage fluid findings of midkine‐deficient (*Mdk* KO;* n* = 19–43) and wild‐type mice (WT;* n* = 21–32) at 7 (A) and 14 (B) days after bleomycin intratracheal administration was compared using the Mann–Whitney *U* test. *P < 0.05 versus *Mdk* KO.

**Figure 6:**
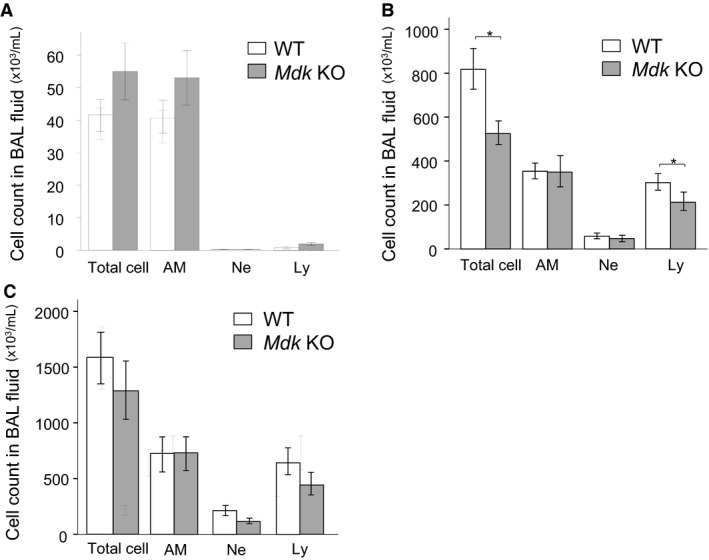
Bronchoalveolar lavage fluid findings before and at 7 and 14 days after intratracheal bleomycin administration. Bronchoalveolar lavage fluid findings of midkine‐deficient (*Mdk* KO;* n* = 4, and 19 for before and after bleomycin administration) and wild‐type mice (WT;* n* = 5, and 17–21 for before and after bleomycin administration) before (A) and at 7 (B) and 14 (C) days after bleomycin intratracheal administration was compared using the Mann–Whitney *U* test. AM, alveolar macrophage, Ne, neutrophil, Ly, lymphocyte. *P < 0.05 versus *Mdk* KO.

With the aim of elucidating the mechanism of the suppression of bleomycin‐induced pulmonary fibrosis in the *Mdk* KO mice, we compared the BAL fluid findings in the *Mdk* KO and WT mice at 7 and 14 days after bleomycin administration. Analysis of the BAL fluid findings showed that total protein concentration at 7 and 14 days (Fig. 5) as well as total cell count or the percentage of lymphocytes at 14 days (Fig. 6) were significantly lower in the *Mdk* KO mice. There were no difference in BAL findings between the *Mdk* KO and WT mice before bleomycin administration (Fig. 6).

### Analysis of inflammatory and fibrotic mediators

**Figure 7:**
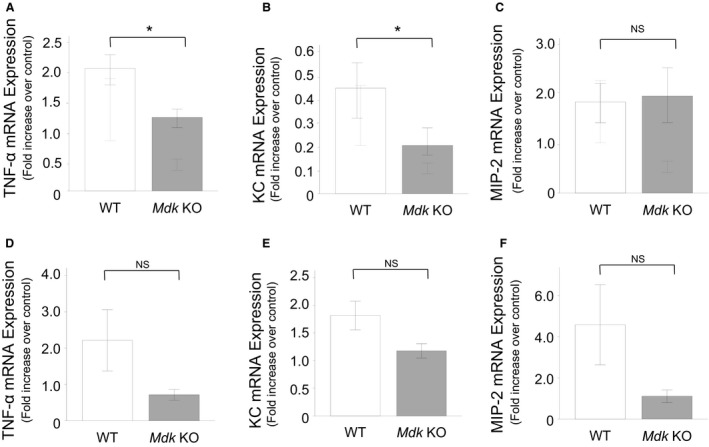
Expression of inflammatory mediators in lung tissue after bleomycin intratracheal administration. Expression of TNF‐*α* (A and D), KC (B and E) and MIP‐2 (C and F) mRNA in the lung tissue of midkine‐deficient (*Mdk* KO;* n* = 9–13) and wild‐type (WT;* n* = 10–12) mice at 7 (A–C) and 14 (D–F) days after bleomycin intratracheal administration was compared using the Mann–Whitney *U* test. NS: not significant. *P < 0.05 versus *Mdk* KO.

We compared the expression of inflammatory and fibrotic mediators in lung tissue in the *Mdk* KO and WT mice. At 7 days after bleomycin administration, the mRNA expression of both TNF‐*α* and keratinocyte chemoattractant (KC), but not of macrophage inflammatory protein 2 (MIP‐2) was lower in the *Mdk* KO compared with the WT mice. At 14 days, there was no difference in the mRNA expression of TNF‐*α*, KC or MIP‐2 between the *Mdk* KO and the WT mice (Fig. 7).

**Figure 8:**
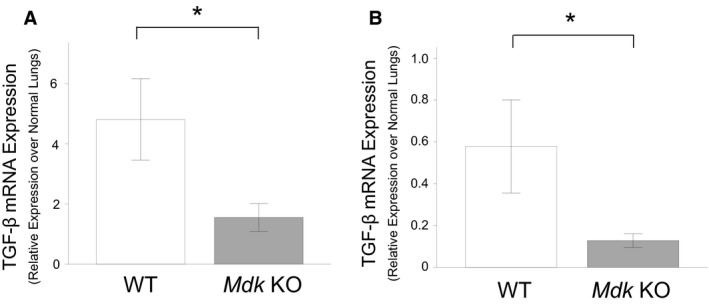
Expression of TGF‐*β* in lung tissue after bleomycin intratracheal administration. Expression of TGF‐*β* mRNA in the lung tissue of midkine‐deficient (*Mdk* KO;* n* = 8–11) and wild‐type (WT;* n* = 7–10) mice at 7 (A) and 14 (B) days after bleomycin intratracheal administration was compared using the Mann–Whitney *U* test. *P < 0.05 versus *Mdk* KO.

We next analyzed the mRNA expression of TGF‐*β*, a growth factor that plays a key role in pulmonary fibrosis. At both 7 and 14 days, the mRNA expression of TGF‐*β* in lung tissue was significantly lower in the *Mdk* KO than in the WT mice (Fig. 8).

## Discussion

This study found that the serum midkine concentration was higher in IPF patients than in healthy subjects, and that the midkine concentrations in the serum and the BAL fluid correlated positively with the percentage of inflammatory cells in the BAL fluid. Also, the expression of midkine was increased in mouse lung tissue after bleomycin administration, while expression of collagen and *α*‐SMA and the pathological lung fibrosis score as well as TNF‐*α* and TGF‐*β* was lower in the lung tissue of *Mdk* KO mice, suggesting the involvement of midkine in the development of pulmonary fibrosis.

IPF is a devastating lung disease of unknown cause and its prognosis is reported to be worse than those of various malignant tumors, excluding lung cancer and pancreatic cancer (Vancheri et al. 2010). Although the mechanisms underlying progression of lung fibrosis have not been fully clarified (American Thoracic Society, 2000), recent evidence suggests the possible involvement of excessive apoptosis and epithelial‐mesenchymal transition (EMT) of alveolar epithelial cells induced by genetic factors (Fukuhara et al. 2013) such as *MUC5B* (Seibold et al. 2011) and telomere‐related genes (Armanios et al. 2007; Tsakiri et al. 2007), and repeated DNA damage by excessive reactive‐oxygen species (Kinnula et al. 2005) in the development of pulmonary fibrosis. In addition, accelerated proliferation and overproduction of ECM components such as collagen due to activation of fibroblasts are strongly involved in the pathogenesis of IPF (Strieter and Mehrad 2009; Nikaido et al. 2015).

In this study, serum midkine was increased in the IPF patients compared to healthy controls, and the midkine concentrations in the serum and the BAL fluid correlated positively with the percentage of inflammatory cells in the BAL fluid. These are consistent with previous reports showing the important role of midkine on inflammation in patients with Crohn's disease, sepsis, and rheumatoid arthritis (Takada et al. 1997; Maruyama et al. 2004; Krzystek‐Korpacka et al. 2010, 2011). Our present study results, generated in IPF patients, similarly suggest the importance of midkine in pulmonary inflammation. There is a considerable debate regarding the role of inflammation in lung fibrosis, especially in IPF (Gross and Hunninghake 2001; Fujimoto et al. 2015). However, an increase in inflammatory cells such as neutrophils in the BAL fluid was reported to be a predictive factor for a poor prognosis for IPF (Rudd et al. 1981; Xaubet et al. 1998) and scleroderma lung disease (Behr et al. 1996). This finding suggests that inflammation is involved in the pathogenesis of pulmonary fibrosis, and that midkine may be involved in fibrotic process via pulmonary inflammation.

In our present study conducted in the mouse bleomycin‐induced pulmonary fibrosis model, midkine expression was increased in lung tissue after bleomycin administration. Increased expression of midkine at sites of tissue injury has been reported (Ohta et al. 1999) and is thought to play an important role in tissue inflammation and repair (Muramatsu 2002). We have shown that expression of collagen and *α*‐SMA and the pathological lung fibrosis score were significantly lower in the lungs of *Mdk* KO compared with WT mice. Those findings indicate that midkine has a profibrotic property, which is in agreement with earlier findings generated in a mouse hyperglycemic nephrosclerosis model (Kosugi et al. 2007) and a heart pressure load model (Natsuizaka et al., 2014).

As a mechanism for suppression of pulmonary fibrosis in *Mdk* KO mice, the suppressed expression of TNF‐*α* and TGF‐*β* in the lung tissue of *Mdk* KO mice was possibly involved in the pathogenesis of pulmonary fibrosis. Although TNF‐*α* is widely accepted as an important proinflammatory cytokine, its profibrotic property is also reported (Postlethwaite and Seyer 1990; Battegay et al. 1995; Sime et al. 1998; Ortiz et al. 1999). TGF‐*β* is known to be an important factor in pulmonary fibrosis (Munger et al. 1999; Zhao et al. 2002; Willis and Borok 2007). Fibroblast proliferation and migration as well as ECM production were promoted by TNF‐*α* and TGF‐*β*. In addition, TGF‐*β* promotes the differentiation of fibroblasts into myofibroblasts (Willis and Borok 2007) and induces epithelial cell apoptosis as well as EMT. Yamada et al. (1997) reported that midkine enhanced TGF‐*β* expression in fibroblasts indicating the strong relationship between these two molecules. There is also the possibility that midkine itself is directly involved in pulmonary fibrosis. Midkine was reported to promote fibroblast proliferation (Kojima et al. 1995) and enhance the synthesis of collagen, glycosaminoglycan and other ECM components (Muramatsu and Muramatsu 1991; Yamada et al. 1997; Sumi et al. 2000). In human keratinocytes, midkine binds to Notch2 and activates the Jak2/Stat3 pathway (Huang et al. 2008), while in A549 human alveolar epithelial cells under hypoxic conditions midkine expression is induced by activation of hypoxia inducible factor‐1*α* via a PKC‐*δ* dependent pathway, and it then promotes EMT (Zhang et al. 2014). It has also been reported that, in WI‐38 human fibroblasts, midkine activates, via anaplastic lymphoma kinase, cellular signals such as phosphoinositide 3‐kinase and MAPK (Stoica et al. 2002) that play important roles in pulmonary fibrosis. Furthermore, Zhang et al. (2015) recently showed that binding of midkine (whose expression was increased by Nox1) to Notch2, and an increase in angiotensin II due to activation of angiotensin‐converting enzyme downstream therefrom, are important for EMT due to mechanical stretch of pulmonary epithelial cells. They also reported that midkine plays an important role in fibrosis after ARDS. These mechanisms may be involved in suppression of pulmonary fibrosis in *Mdk* KO mice.

In this study, we demonstrated that total protein concentration, the total number of cells and the percentage of lymphocytes in the BAL fluid were lower in *Mdk* KO mice than in WT mice, showing midkine had an anti‐inflammatory effect in this model. The results of this study do not elucidate the details of why inflammation was suppressed in the *Mdk* KO mice. However, midkine was reported to be involved in the expression of chemokines such as monocyte chemoattractant protein‐1 and MIP‐2 (Sato et al. 2002), and also to be involved in adhesion of inflammatory cells to vascular endothelial cells via *β* integrin (Herter and Mayadas 2014; Weckbach et al. 2014). These mechanisms may thus be involved in midkine‐mediated inflammation. Because, in bleomycin pulmonary fibrosis model, inflammation precedes and affects sequential fibrosis, it is possible that the suppression of inflammatory attenuated pulmonary fibrosis in *Mdk* KO mice. On the other hand, it is interesting that time course of midkine expression in the lungs was biphasic after bleomycin instillation. Increased expression was found in the early inflammatory stage (day 3) and again in the late fibrosing stage (day 14 and 21). Because midkine has both inflammatory and fibrotic properties, lack of midkine might have dual effects (anti‐inflammatory and anti‐fibrotic effects) in *Mdk* KO mice.

This study has some limitations. First, we could not find the correlation between the levels of midkine and physiologic parameter in patients with IPF. Although we cannot explain the exact reason, it is possible that we could not detect the correlation because the analysis was performed at just one time point. Because IPF is a heterogeneous disorder with a variable clinical course, rapid progression occurs in some IPF patients and we cannot find out any change in others. Furthermore studies are necessary to determine the role of midkine in patients with IPF by evaluating the relationship between midkine levels and prognosis. Second, the mean age was significantly lower in healthy volunteers compared to patients with IPF. Because the relationship between age and levels of midkine has not yet been investigated in healthy individuals, we must await further studies to clarify whether age affects the levels of midkine. Third, we could not evaluate the precise role(s) of midkine on pulmonary fibrosis in bleomycin model. Further experiments using other pulmonary fibrosis models and in vitro experiments are necessary to evaluate the role of midkine on pulmonary fibrosis in detail.

Taken together, however, the results of this study indicate that midkine is potentially involved in the development of pulmonary fibrosis by regulating inflammatory cell migration into the lung and expression of TNF‐*α* and TGF‐*β*.

## Conflict of Interest

The authors declare that no conflict of interest exists.
